# Observing short-range orientational order in small-molecule liquids

**DOI:** 10.1038/s41598-022-27187-7

**Published:** 2022-12-28

**Authors:** Anton Gradišek, Tomaž Apih, Maria J. Beira, Carlos Cruz, Susete N. Fernandes, Helena M. Godinho, Pedro J. Sebastião

**Affiliations:** 1 Jožef Stefan Institute, Jamova Cesta 39, 1000 Ljubljana, Slovenia; 2 Center of Physics and Engineering of Advanced Materials, Instituto Superior Técnico, Universidade de Lisboa, Av. Rovisco Pais, 1049-001 Lisboa, Portugal; 3 Department of Physics, Instituto Superior Técnico, Universidade de Lisboa, Av. Rovisco Pais, 1049-001 Lisboa, Portugal; 4 CENIMAT/I3N, Departamento de Ciência Dos Materiais, Faculdade de Ciências E Tecnologia, UNL, 2829-516 Caparica, Portugal

**Keywords:** Structure of solids and liquids, Liquid crystals

## Abstract

Local molecular ordering in liquids has attracted a lot of interest from researchers investigating crystallization, but is still poorly understood on the molecular scale. Classical nucleation theory (CNT), a macroscopic thermodynamic description of condensation, has shortcomings when dealing with clusters consisting of tens of molecules. Cluster formation and local order fluctuations in liquid media are difficult to study due to the limited spatial resolution of electron- and photon-imaging methods. We used NMR relaxometry to demonstrate the existence of dynamic clusters with short-range orientational order in nominally isotropic liquids consisting of elongated molecules. We observed clusters in liquids where the local ordering is driven by polar, steric, and hydrogen-bond interactions between the molecules. In the case of a liquid crystal, measuring the local orientational order fluctuations allowed us to observe the size of these clusters diverging when approaching the phase transition from the isotropic to the nematic phase. These fluctuations are described in terms of rotational elasticity as a consequence of the correlated reorientations of the neighbouring molecules. Our quantitative observations of the dynamic clusters in liquids, numbering about ten or fewer molecules, indicate that this is a general phenomenon in various types of liquids.

## Introduction

Nucleation plays a crucial role in phase transitions from a disordered to a more ordered phase, as in the case of gas to liquid, liquid or solution to solid, isotropic to nematic phase in liquid crystals, or even in the phase transitions of magnetic systems. The underlying framework of classical nucleation theory (CNT), the most common theoretical approach to studying diverse systems such as rain formation, the aggregation of metallic clusters from a melt or a solution, as well as the aggregation of organic molecules, including protein crystallization^1–5^, has been in use since at least the 1970s^1^.

However, CNT does not discuss the nucleation process at the level of individual molecules, a topic that the refinement of experimental techniques is now opening to further studies. Both theoretical and experimental results are providing support to alternative nucleation pathways, such as multi-step nucleation or pre-nucleation cluster formation^1,6,7^. Because clusters involve a small number of molecules (down to the shell of nearest neighbours, which is about ten molecules or fewer), measuring their collective fluctuations is extremely challenging in an experiment. Wave-imaging methods, such as transmission electron microscopy (TEM), were used to study cluster formation on some model systems in planar geometries^3,8^. However, these methods lack the temporal and spatial resolution to provide an insight into the thermal fluctuations and the general behaviour of molecular clusters, both in bulk liquids and in the subcritical region^3,6^.

A common view of isotropic liquids (including liquid crystals above the clearing point^9^ assumes no positional and orientational order. However, in the case of non-spherical building blocks (molecules) of the liquid, some degree of local orientational ordering is nevertheless expected to occur. In liquid crystals, the presence of correlated motions in so-called nematic cybotactic clusters in the isotropic phase was hinted at by nuclear magnetic resonance (NMR) relaxation and spectra^10–16^ as well as optical^17,18^ measurements back in the 1960s and 70s. More recently, molecular dynamics simulations have indicated the existence of local ordering at several degrees above the clearing temperature^19–21^. The fluctuations in liquids have been studied with light-scattering methods, mostly focusing on very specific systems such as supercooled liquids or mixtures forming aggregates^22^. Improved dynamic light scattering and scanning electron transmission methods can typically achieve resolution limits of the order of 2–7 nm or better when accessing solid particle aggregates^23,24^.

Here we show that orientational short-range ordering is a feature of a heterogeneous group of liquids consisting of elongated molecules with different types of intermolecular interactions, and that it can be analysed with a common approach. We demonstrate this for cases of molecules with polar interactions (5CB, 4′-pentylbiphenyl-4-carbonitrile-α-d_2_, as a model compound that exhibits a liquid crystalline phase), hydrogen bonds (hexanoic acid), or purely steric ordering (2-methyl-1-undecene). The analysis of local ordering is performed in terms of molecular dynamics accessed by means of NMR relaxometry over a frequency range spanning five orders of magnitude of Larmor frequency (ν_L_, the frequency of precessing nuclear magnetic moments, which is directly proportional to the external magnetic field) by combining conventional and fast field-cycling relaxation techniques, diffusometry, and X-ray diffraction (experimental details are in the Supplementary Information). Short-range fluctuations of orientation within the clusters are modelled by taking into account the viscosity and rotational elasticity, the latter being a phenomenon where a molecule experiences torques from the surrounding molecules, restricting its rotations/reorientations^9,25^. On the other hand, X-ray diffraction provides information about the molecular stacking. The combination of two approaches allows us to determine the correlation length of the clusters that can be seen as an initial phase of nucleation.

The proton in a hydrogen atom has spin angular momentum and an associated magnetic moment. Because its motion can be perturbed by magnetic forces resulting from neighbouring spins, the spin dynamics of a proton is sensitive to a change in the separation of nearby spins and their orientation with respect to an external magnetic field. The excited proton spins will relax to equilibrium along the external magnetic field with a characteristic longitudinal relaxation time T_1_^26^.

Looking at the relaxation of proton spin magnetization on a molecule can provide information about the motions of individual molecules, such as rotations/reorientations (R) and translational self-diffusion (SD), as well as of the correlated molecular reorientations of groups of molecules. In the case of elongated molecules, R can take place about the long or short molecular axis. Collective motions are correlated over a distance that is usually referred to as the coherence length. Dynamic processes take place on different time scales, ranging from picoseconds for individual motions to milliseconds for collective motions in ordered phases of liquid crystals. The measured total spin-relaxation rate is a sum of the contributions of the relaxation mechanisms due to the above-mentioned motions, as they are statistically independent, owing to different timescales and/or because these motions are not correlated^25^. Longitudinal relaxation measurements at a given Larmor frequency ν_L_ are most sensitive to motions with characteristic times, also referred to as correlation times, of the order of 1/ν_L_. Therefore, measuring the longitudinal relaxation rate (1/T_1_) over a broad range of ν_L_ (5 kHz to 300 MHz) allows us to probe the temporal and spatial characteristics of all the dynamic processes mentioned above^27^.

## Results and discussion

**Figure 1:**
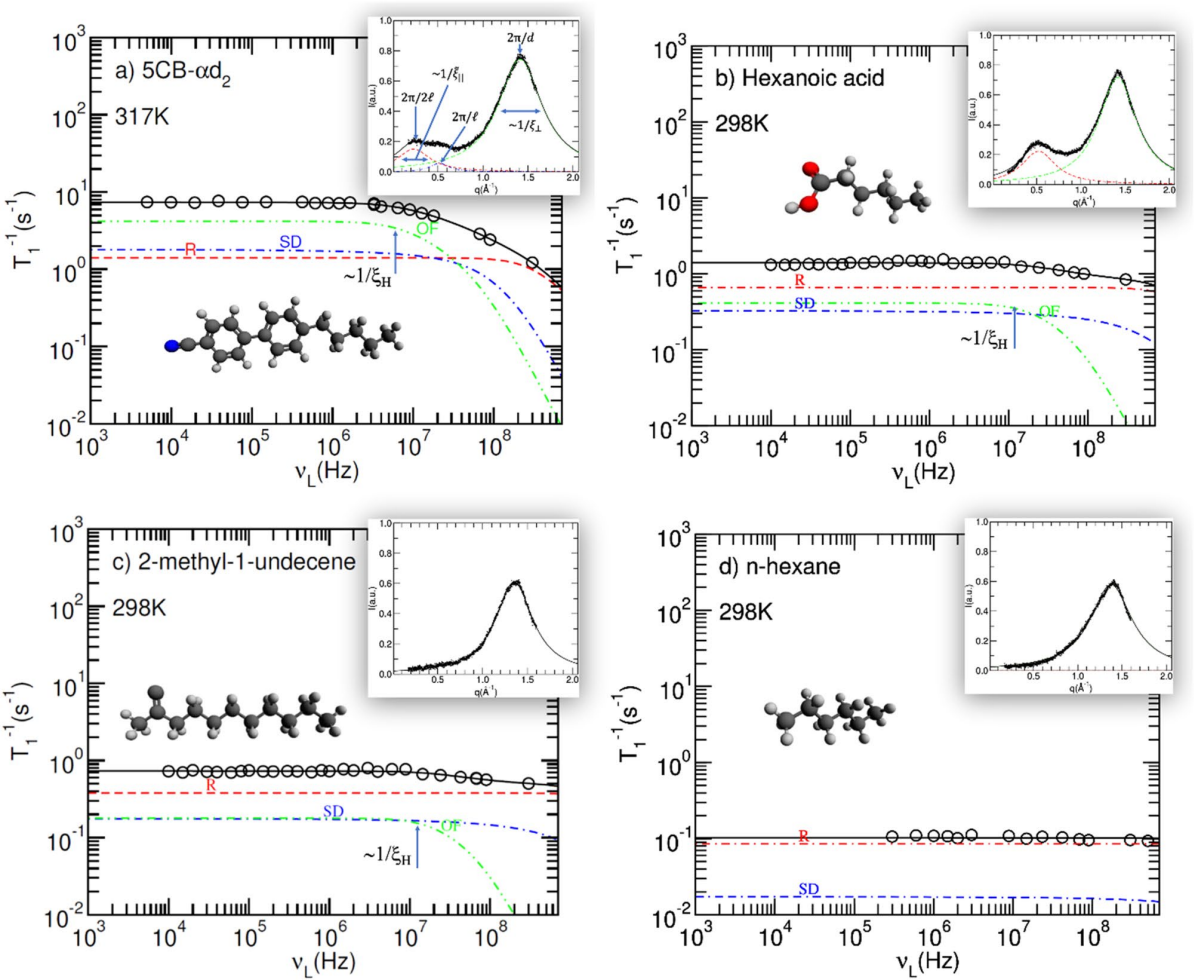
Proton longitudinal relaxation rate as a function of Larmor frequency for 5CB (**a**), hexanoic acid (**b**), 2-methyl-1-undecene (**c**), and n-hexane (**d**), together with the corresponding model fits. Contributions of individual relaxation mechanisms are shown separately (red dash: rotations/reorientations, R; blue dot-dash: self-diffusion, SD; and green double dot-dash: order fluctuations, OF), and as a sum (solid black line). Insets show the X-ray diffraction profiles at the corresponding temperatures, accompanied by the model fits*.*

Figure 1 shows analyses of the relaxation data for the four systems studied. In all cases the relaxation rate T_1_^−1^ reaches a plateau at low frequencies, with the values decreasing from system (a) to (d). The more correlated the local motions are, the less averaging out of fluctuations of spin interactions takes place in the low-frequency range, resulting in a higher relaxation rate. This is the most prominent in 5CB, where the molecules are the most prone to spontaneous orientation. On the other hand, above 10 MHz, the dispersion curves are different, with the relaxation rate prominently decreasing for 5CB, while remaining flat for n-hexane. The cut-off frequency, below which the T_1_^−1^ values become frequency independent, can be directly related to the length scale of the local order in each system. On the other hand, local ordering can also be seen from the X-ray diffraction. The diffraction profiles are shown as insets for each system. For all the systems, the strongest peak corresponds to the nearest-neighbour distance in a lateral direction. An additional peak at lower *q* values is observed for the 5CB and hexanoic acid, indicating additional molecular stacking, but it is absent for the other two systems. The analyses of the relaxation dispersion and of the diffraction profile thus point to the existence of local ordering in all the systems except n-hexane.

In a purely isotropic liquid we would expect two dynamic processes: molecular rotations/reorientations and translational self-diffusion. At this point, let us focus on the system that has the most rigid core, i.e., the 5CB. A self-consistent, theoretical analysis of relaxation data at several temperatures deep into the isotropic phase of 5CB shows that the proton relaxation cannot be described solely using these two mechanisms. These models consider the microscopic characteristics of the building blocks (size, shape, and typical inter-molecular distances, accessible through X-ray diffraction) and macroscopic ones (visco-elastic properties), as well as diffusion, which we measured or obtained from previous studies of the system^28–30^. The order of magnitude of the contribution of the rotations/reorientations of individual molecules is constrained by the deuterium NMR results presented in previous studies^31,32^. Here, we propose that the additional required mechanism is associated with collective motions inside the short-lived cybotactic clusters^9,25^ that form within the isotropic phase (order fluctuations). For this mechanism, the only free model parameters are the correlation times τ and the coherence length ξ of the molecular motions in the clusters.

**Figure 2:**
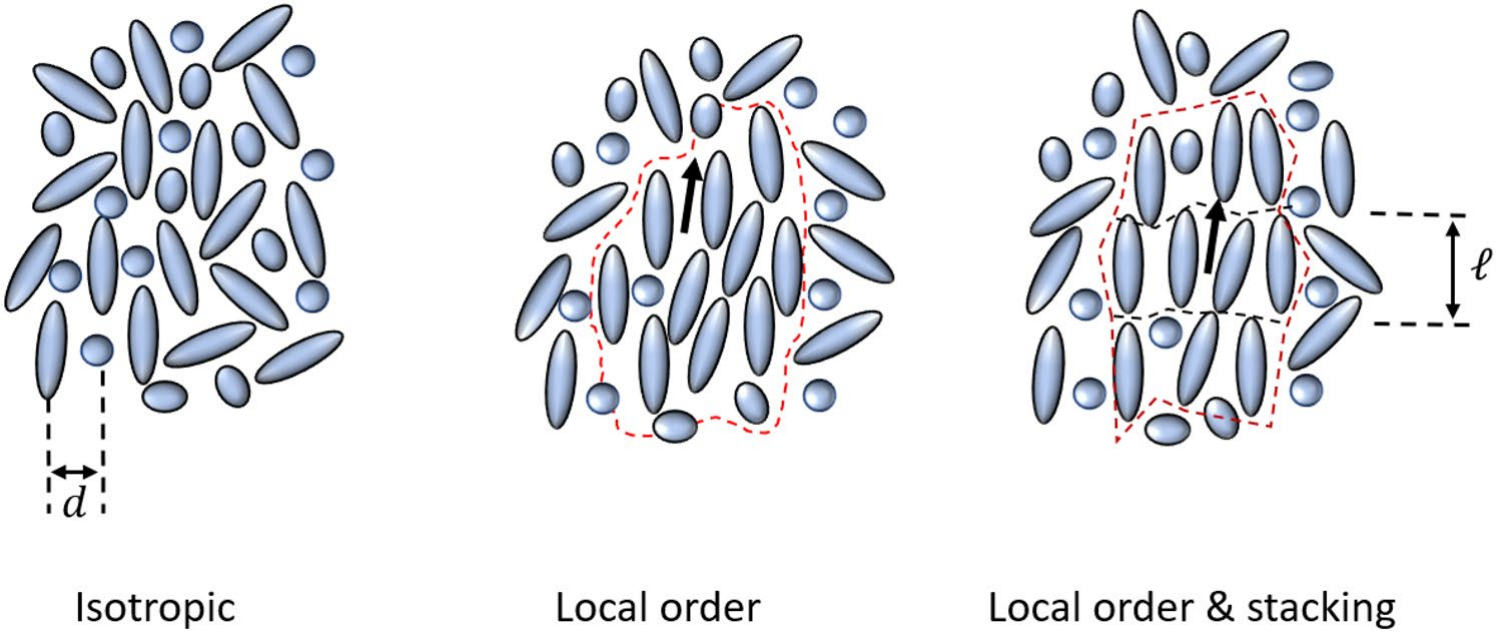
Schematic representations of the short-range local organization of molecules in an isotropic liquid: purely isotropic, clusters with local orientational order but without stacking, and clusters with some degree of stacking. The red dashed line is a guide to an eye and should not be seen as a cluster boundary*.*

These clusters are regions where the rod-like molecules are temporarily aligned (as illustrated in Fig. 2) and the tumbling of one molecule inherently influences the movements of the aligned neighbours. This phenomenon is linked to both the rotational viscosity and the rotational elasticity in the system. The inset in Fig. 1a shows two broad X-ray diffraction peaks, the high scattered wave vector *q* peak corresponding to the lateral distance *d* between the molecules, while the low-*q* ones relate to longitudinal molecular stacking with a periodicity of a single or double molecular length *l*. The full-width at half-height of each peak indicates that the molecules are stacking in each direction to the nearest neighbours, from which the coherence lengths ${\xi }_{||}$ and ${\xi }_{\perp }$, and, therefore, the size of the molecular clusters can be estimated. These clusters can be described using the formalism developed by de Gennes^9^ and Dong^25^ for pre-transitional effects in LCs. In these clusters, fluctuations of the order parameter depend on the rotational viscosity and the rotational elasticity, while the coherence lengths of these fluctuations match the cluster size. Considering all the independently obtainable physical parameters of the system, we can consistently describe all the experimental data and estimate both the local rotational elasticity and the coherence length ${\xi }_{||}$, which in the case of NMR measurements we denote by ${\xi }_{H}$ and is close to ${\xi }_{||}$ as the magnetic contribution to the ordering is small. This value is associated with the low-frequency plateau of the relaxation profile for this mechanism (Fig. 1a). A comprehensive analysis of the temperature dependence from more than 10 K above the clearing point down to the nematic phase of 5CB is presented in the Supporting information.

In contrast to liquid crystals, hexanoic acid, 2-methyl-1-undecene and n-hexane do not exhibit any isotropic-ordered liquid-phase transition. For these non-liquid crystalline liquids we have readily available interatomic distances, self-diffusion constants, and visco-elastic properties, allowing us to conduct the same type of molecular dynamics analysis. These three liquids were studied at room temperature, which is far from their respective freezing points. Interestingly, for both the hexanoic acid (Fig. 1b) and the 2-methyl-1-undecene (Fig. 1c), the experimental results cannot be explained without taking the effects of collective motion into account, but they can be for n-hexane (Fig. 1d). For these liquids, any local ordering of the constituent molecules cannot be associated with pre-transitional effects only. Looking at the structure, the hexanoic acid molecules are asymmetric, with a carboxylic group that allows it to form hydrogen bonds and induce longitudinal stacking. On the other hand, the 2-methyl-1-undecene molecules are also asymmetric, but contain no groups that would promote local lateral coordination, and thus stacking. Still, these long molecules can locally align. As a consequence, the X-ray diffraction profile of the hexanoic acid exhibits a low-q peak associated with longitudinal ordering, but that peak is absent in the 2-methyl-1-undecene because there is no periodicity along the long axis. Finally, the NMR relaxation data of the n-hexane can be described completely using only the rotations/reorientations and the self-diffusion mechanisms as the molecular aspect ratio seems to be too small to promote local alignment. Consistently, there is no low-q peak in the X-ray diffraction profile (Fig. 1c,d). Schematic representations of all three cases are shown in Fig. 2.

**Figure 3:**
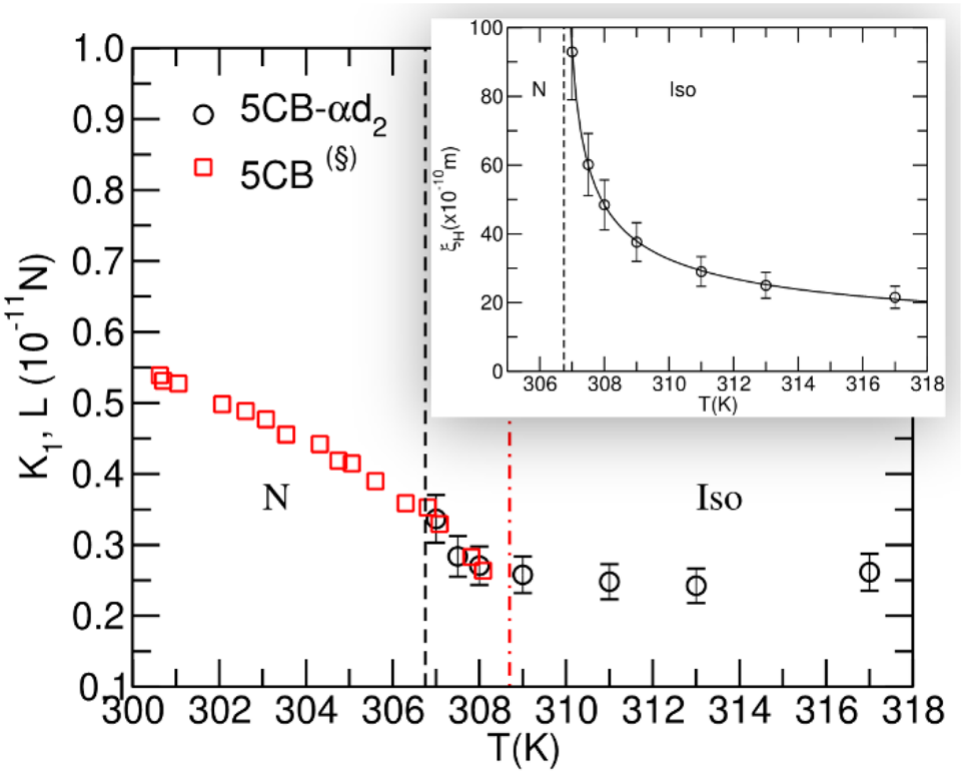
Values of Frank’s elastic constant K_1_ for 5CB as a function of temperature. In the nematic phase (red squares) the values were obtained from Bunning et al.^30^ using magneto-optic techniques to study the Fréedericksz transition. The values of rotational elasticity L in the isotropic phase (black circles) of 5CB-$\mathrm{\alpha }$ d_2_ were obtained from the NMR data analysis. The inset figure shows the coherence length as a function of temperature and the model fitting curve ${\upxi }_{H}=A/\sqrt{T-{T}_{c}^{*}}+{\upxi }_{\infty }$.

For all four systems, we estimated the amplitude of the rotational elasticity L in the local clusters. For the 5CB system, the temperature analysis of the NMR relaxation data allows us to directly compare the values of the rotational elasticity in the isotropic phase with the values of Franck’s splay elastic constant (K_1_)^9^ reported for this system by Bunning et al.^30^ in the nematic phase where long-range order exists, and distortions of the average molecular alignment are observed. As seen in Fig. 3, there is a match where the two overlap around the phase transition. The implication is that the isotropic phase can be seen as “dynamically elastic” in view of the elasticity of the ordered clusters, which is also in line with the concept of “rigidity” introduced by P. W. Anderson with the breaking of the symmetry^33,34^. If a liquid is disordered, as is the common understanding, it cannot convey static forces or torques, but can convey them during the lifetime of the clusters.

Close to the phase transition, the ordered domains grow and the nematic order starts to set in, known as the pre-transitional effect. A few degrees above the transition, there is an overlap between the rotational elasticity, as a feature of the local domains, and the elasticity associated with distortions of the nematic director field that sets in the continuous medium. On the other hand, far above the transition, the rotational elasticity appears to be constant.

The inset in Fig. 3 shows the critical behaviour of the coherence length of the fluctuations that matches the predictions of the first-order isotropic-nematic phase transition in a LC, as developed by de Gennes^9^. Approaching the transition, the coherence length diverges, which corresponds to the growth of the clusters that eventually evolve to a continuous nematic phase. At higher temperatures, the coherence length levels off to a plateau in the temperature range analysed, corresponding to clusters consisting of only the nearest neighbours. These experimental observations are consistent with the molecular dynamics simulations^19–21^ and the optical measurements for other liquid crystals^16–18^. The values for other systems are listed in the Supporting Information.

## Conclusions

Our findings not only experimentally confirm and quantitatively evaluate the existence of clusters with local order in a bulk liquid crystal far above the clearing point, but furthermore demonstrate that this phenomenon is present in several liquids consisting of molecules where their geometry and/or inter-molecular interactions promote local alignment. Any evidence of coherent molecular motions in liquids implies the existence of elasticity of the system—otherwise there would be no collective motions. Apart from the spherically symmetrical particles, this phenomenon appears to be independent of molecular shape and of the type of interaction (hydrogen bond, polar, etc.), as long as the lifetime of the clusters is long enough to allow for a distinction between the individual and coherent motions. In terms of crystallization, it serves as an intrinsic self-stabilizing process, as any other organization would have to overcome the elastic torques within the cluster. As we demonstrated, NMR can distinguish between the movements of individual molecules and the coherent motions of molecules within a dynamic assembly. Quantifying these processes has implications for the understanding of crystallization for even larger molecules, including macromolecules such as proteins.

## Supplementary Information

Supplementary Information.

## Data Availability

All data are available in the main text or the supplementary materials.
