# Supplementary material for: Observing short-range orientational order in small-molecule liquids
Source: Sci Rep. 2022 Dec 28;12:22500. doi: 10.1038/s41598-022-27187-7 (PMC9797480; doi:10.1038/s41598-022-27187-7)
Supplement: Supplementary file 1 — Supplementary Information. [file 41598_2022_27187_MOESM1_ESM.pdf]

Supplementary Materials for

Observing short-range orientational order in small-molecule liquids

Anton Gradišek*, Tomaž Apih, Maria J. Beira, Carlos Cruz, Susete N. Fernandes, Helena M. Godinho, Pedro J. Sebastião*

Correspondence to: anton.gradisek@ijs.si, pedro.jose.sebastiao@tecnico.ulisboa.pt

Materials

Figure S1 shows the molecular structures of all four systems studied. The α-deuterated liquid crystal 4'-pentylbiphenyl-4-carbonitrile-α-d_2 (5CB$-\alpha d_2$) was obtained as described in *(29)*. The other three compounds were bought from Sigma-Aldrich.

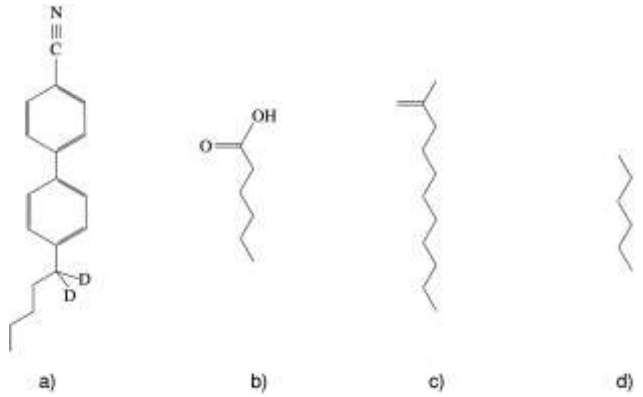

Figure S1: Molecular structures of the studied compounds: a) 4'-pentylbiphenyl-4-carbonitrile (5CB$-\alpha d_2$); b) hexanoic acid ($C_6H_{12}O_2$); c) 2-methyl-1-undecene ($C_{12}H_{24}$); d) n-hexane (C_6H_{14}).

Methods

The spin-lattice relaxation dispersion data were acquired in three different setups. Proton relaxation rates at Larmor frequency of $\nu_L(^1H)$ = 300 MHz, corresponding to B = 300 MHz $\times\, 2\pi/\gamma_H$= 7.05 T, where γ_H is the proton gyromagnetic ratio, were measured using a Bruker superconducting magnet. Relaxation rates at 90 and 67 MHz were measured using a Bruker BE-30 electromagnet. In both setups, the inversion recovery sequence was used to measure spin-lattice relaxation. Relaxation rates in the Larmor frequency range from 18 MHz to 5 kHz were measured using a fast field-cycling relaxometer SPINMASTER FFC-2000 (Stelar, s.l.r.). To measure the relaxation rates, the non-polarized (NP) sequence was used above 5 MHz and the pre-polarized (PP) sequence below this value. For the PP sequence, the polarization frequency was 18 MHz, corresponding to 0.42 T. The acquisition frequency in both sequences was 9.25 MHz. All other parameters were optimized according to each experiment.

For hexanoic acid, 2-methyl-1-undecene, and n-hexane the spin-lattice relaxation measurements in the frequency range 10 kHz-9 MHz were conducted using a home-built fast field-cycling relaxometer *(35)*.

In addition, for these samples the translational self-diffusion coefficient was measured by PFG NMR using a pulsed gradient stimulated echo sequence, a Bruker Diff 30 probe, a magnetic field gradient unit and a Bruker 7 T superconductor connected to a Bruker Avance III NMR console. The ^{1}H diffusion coefficient of n-hexane was later determined using a 500 MHz superconducting magnet, also paired with a Bruker Avance III console.

In all three setups, the 5CB$-\alpha d_2$ sample was first heated 30 K above the N-I transition and then slowly cooled with a cooling rate ≤ 1 K/min to the desired temperature. Temperature was controlled with a standard gas-flow system with a precision of ± 0.2 K.

X-ray profiles were obtained at controlled temperature using the powder method with 1mm rotating capillaries. The experiments were performed using a computer-controlled data acquisition system paired with an INEL CPS 590 gas curved counter on a variable-geometry setup equipped with a Max-Flux Optic graded multilayer monochromator for CuKα radiation, λ = 1.54056 Å. The apparatus acquisition system was calibrated using the scattering peaks of a Silver Behenate sample. The sample oven temperature was calibrated monitoring the diffractograms' changes at the known transition temperatures of the liquid crystal 9O.4 [4-(n-nonyl)oxybenzylidene-4-(n-butyl)aniline] (*36)*. The background noise was eliminated by subtraction of X-ray profiles obtained on an empty 1 mm capillary tube. Channel offset corrections were made, and the good sample alignment allowed for positive-negative data averaging.

Experimental results

The experimental results for the spin-lattice relaxation rate (T_1^{-1}) as a function of Larmor frequency and temperature for 5CB$-\alpha d_2$ are presented in Figures S2 and S3, respectively. At high fields, T_1^{-1} changes little with temperature, while it gets considerably longer and temperature-dependent at lower fields, below the MHz range. The I-N phase transition takes place between 307 K (still isotropic) and 306.5 K (already nematic), what is reflected in the change of the shape of the dispersion curve. The dispersion curve at 307 K shows a clear deviation from the typical low-frequency plateau, characteristic of an isotropic phase, which indicates a biphasic region (See Figures S2 and S3).

In view of the fact that we are dealing with a bulk sample and that the precision of the temperature controller is limited, we avoided more detailed measurements around the phase transition temperature, as temperature gradients in the sample may influence the exact transition point.

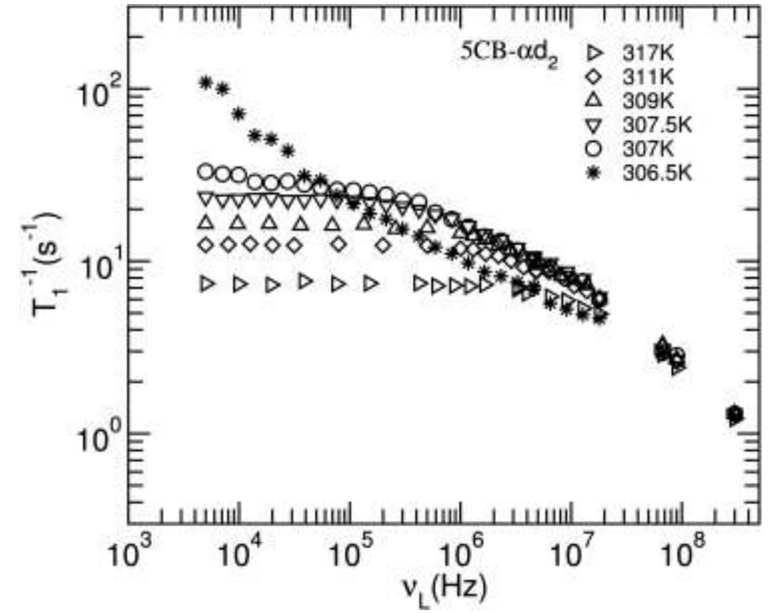

Figure S2: 5CB−αd$_2$ spin-lattice relaxation dispersions in the isotropic phase at 317, 311, 309, 307.5, and 307 K, and in the nematic phase at 306.5 K

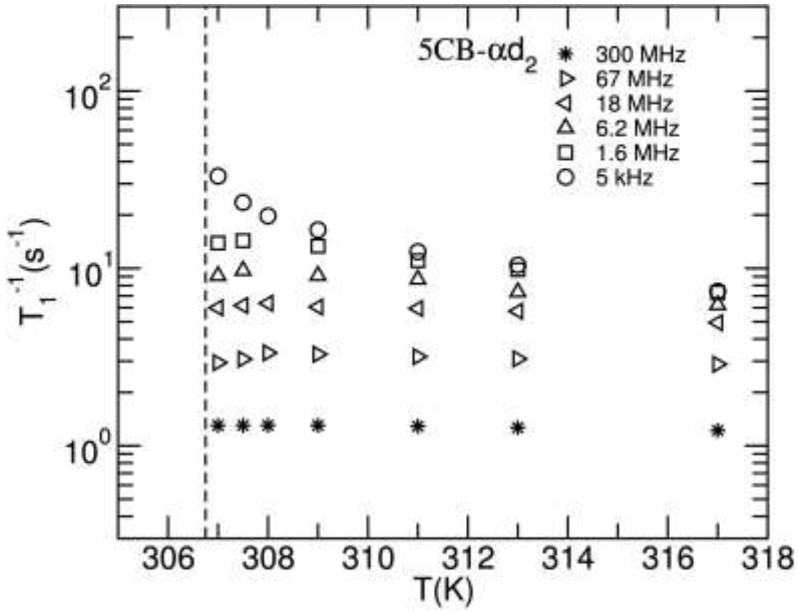

Figure S3: Temperature dependence of T_1^{-1} for 5CB−αd$_2$ in the isotropic phase at some selected Larmor frequencies

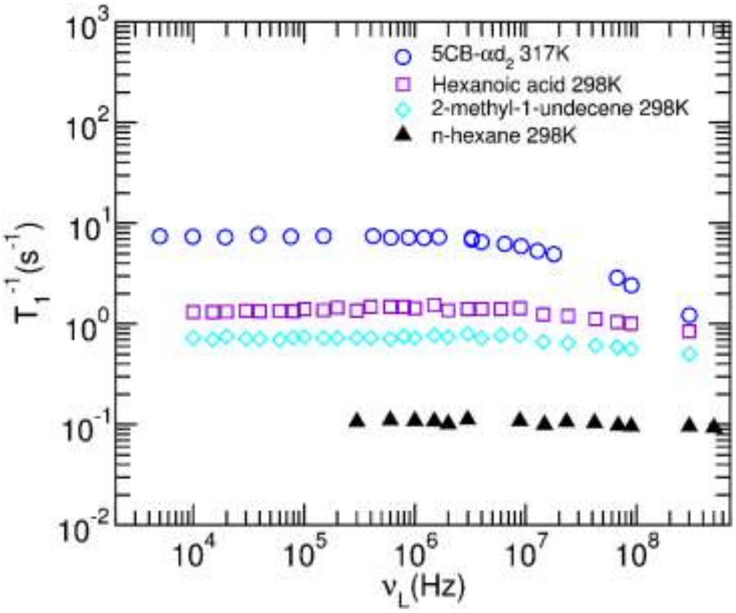

Figure S4: Spin-lattice relaxation dispersions of 5CB$-\alpha$d2 in the isotropic phase at 317 K and of 2-methyl-1-undecene, hexanoic acid, and n-hexane at 298K.

Relaxation mechanisms

Proton spin-lattice relaxation is governed by fluctuations of dipolar spin interactions, both homonuclear and heteronuclear. Here, we will only focus on H-H dipolar interactions as all heteronuclear interactions have been observed to be negligible in comparison *(25,28,29)*. Molecular movements cause the distances between spins to vary, as well as the angles between the inter-spin vectors and the external magnetic field. Proton relaxation is influenced both by intramolecular and intermolecular interactions. In liquid crystals, it is often possible to distinguish different types of molecular motions that are either statistically independent or have distinct characteristic correlation times, therefore the total relaxation rate can be expressed as the sum of contributions of individual relaxation processes.

In the isotropic phase, the two dynamic processes that are typically considered in the analysis are molecular rotations/reorientations (R) and translational self-diffusion (SD). In ordered phases, collective motions start to play a role as well. Examples of such collective motions are order director fluctuations (ODF) in the nematic phase, layer undulations in smectic phases, and rotations, mediated by translational diffusion along the helical axis (RMTD) in chiral phases *(25,28,37-39)*. In the isotropic phase close to the phase transition temperature, the appearance of the nematic cybotactic domains also results in collective motions. The coherence length associated with the size of these domains shows critical behaviour when approaching the I-N transition temperature and *short-range director fluctuations* (DF) start to be observed. Far from the phase transition, the size of these domains is limited to the size of two molecules which share a common alignment of the long molecular axis. In this case, we call the mechanism *short-range nematic order fluctuations* (OF).
In the analysis of the spin-lattice relaxation in 5CB$-\alpha d_2$ in the isotropic phase, we consider the following mechanisms:

Translational self-diffusion

Translational self-diffusion (SD) in the isotropic phase of bulk liquid crystals can be calculated analytically using the model of Torrey *(40)*. For protons (^{1}H)

$$(T_1^{-1})_{SD}(\omega_L) = \frac{3}{2} K_{dd}^2 I(I+1) \frac{n\alpha}{Dd} [\mathcal{T}(\alpha, \omega_L) + 4\mathcal{T}(\alpha, 2\omega_L)] \quad \text{(S1)}$$

where $\omega_L = \gamma_H B$, is the Larmor frequency, and γ_H is the proton's gyromagnetic ratio. This model depends on the spin density n, the self-diffusion constant D, the mean square jump distance $\langle r\text{^}2\rangle$, and the width of the molecules d. In addition, the model depends on $\alpha = \langle r^2\rangle/(12d^2)$ that describes the type of diffusion process from $\langle r^2\rangle \sim d^2$ for strong collisions to $\langle r^2\rangle \ll d^2$for small step ("continuous") diffusion. I = 1/2 for ^{1}H nuclear spins and $K_{dd} = \mu_0\gamma_H^2\hbar/(4\pi)$. Function $\mathcal{T}(\alpha, \omega_L)$ can be found in *(40)*.

Single molecular rotations/reorientations

The contributions of molecular rotations/reorientations can be treated by the model developed by Nordio *(25)*. Here,

$$(T_1^{-1})_R(\omega_L) = \frac{3}{2} K_{dd}^2 I(I+1) \left[\mathcal{J}_{\mathcal{R}}^{(1)}(\omega_L) + \mathcal{J}_{\mathcal{R}}^{(2)}(2\omega_L)\right] \quad \text{(S2)}$$

where the rotational correlation functions used to get spectral densities $\mathcal{J}(\omega)$ are here expressed for cylindrical molecules reorienting in uniaxial mesophases. Following Abragam's notation *(26)* ($m_L \neq 0$):

$$\mathcal{J}_{\mathcal{R}}^{(m_L)}(m_L\omega_L) = \frac{4}{3}(m_L)^2 \sum_{m_M=0}^{2} A^{(m_M)} c(m_L, m_M) \frac{\left(\tau_{m_L m_M}^2\right)^1}{m_L^2\omega_L^2 + \left(\tau_{m_L m_M}^2\right)^{-2}} \quad \text{(S3)}$$

with the correlation times τ_{m_L,m_M}^2 given by

$$\left(\tau_{m_L,m_M}^2\right)^{-1} = \frac{\tau_S^{-1}}{\beta_{m_L m_H}^2} + (\tau_L^{-1} - \tau_S^{-1}) m_{m_H}^2 \quad \text{(S4)}$$

while $c(m_L, m_M)$ and $\beta_{m_L m_M}^2$ can be estimated numerically as functions of the nematic order parameter S. The average molecular geometrical factors $A^{(0)} = \overline{\sum_{j\neq i}\left|d_{0,0}^2(\alpha_{kl})\right|^2/r_{kl}^6}$ and $A^{(m_M)} = 2\overline{\sum_{j\neq i}\left|d_{m_M,0}^2(\alpha_{kl})\right|^2/r_{kl}^6}$ for $m_M \neq 0$ were estimated for 5CB-αd_2, assuming the most stretched molecular conformation.

In the isotropic phase the nematic order parameter S is zero and in Eq. (3), $c(m_L, m_M)$ = 1/5 and $\beta_{m_L m_H}^2 = 1/6$. τ_L^{-1} and τ_S^{-1} are the rotational correlation times along the long and short molecular axis, respectively.

Correlated rotations/reorientations and short-range order fluctuations

The short-range order fluctuations have been described in terms of purely viscous relaxing processes with characteristic times, $\tau_q^{-1} = Lq^2/\eta$, where η is the viscosity, L has the dimensions of an elastic constant *(9)*. From the phenomenological point of view, the underlying assumptions of this model should also apply to other liquids.

The contribution to the proton spin-lattice relaxation rate of order fluctuations in these nematic cybotactic domains in the isotropic phase of liquid crystals can be described for ^{1}H spins as

$$\left(\frac{1}{T_1}\right)_{OF} = \frac{3}{2}K_{dd}^2\frac{\overline{\left(3\cos^2\alpha_{ij}-1\right)^2}}{4r_{ij}^6}\frac{k_BT\eta^{1/2}}{4\pi^2L^{3/2}}\int_0^{\omega_c}\frac{\sqrt{x}dx}{\omega_L^2+(x+\omega_0)^2}$$
$$= A_{OF}\int_0^{\omega_c}\frac{\sqrt{x}dx}{\omega_L^2+(x+\omega_0)^2} \qquad \text{(S5)}$$

where $\omega_0 = L/(\eta\xi_H^2)$ and $\omega_c \sim L\pi^2/(\eta\ell^2)$, being ℓ the molecular length. ξ_H is the orientational coherence length and is related to the size of the domains where coherent order fluctuations are observed *(9)*. r_{ij} and α_{ij} are the inter spin distances and angles with respect to the long molecular axis, respectively.

Close to the I-N transition, the size of the cybotactic domains presents a critical behaviour and director fluctuations might be observed in these larger clusters presenting nematic order *(9)*. The contribution of this collective motion can be estimated using the same relaxation model considered for the collective motions in the nematic phase

$$\left(\frac{1}{T_1}\right)_{DF} = \frac{9}{8}K_{dd}^2\frac{\overline{\left(3\cos^2\alpha_{ij}-1\right)^2}}{4r_{ij}^6}\frac{k_BTS^2\eta^{1/2}}{2\pi^{3/2}K^{3/2}}\frac{1}{\sqrt{\nu}}\left[f\left(\frac{\nu_{cM}}{\nu}\right)-f\left(\frac{\nu_{cm}}{\nu}\right)\right]$$
$$= A_{DF}\frac{1}{\sqrt{\nu}}\left[f\left(\frac{\nu_{cM}}{\nu}\right)-f\left(\frac{\nu_{cm}}{\nu}\right)\right] \qquad \text{(S6)}$$

where $\nu_{cM} = 2\pi K/(\ell^2\eta)$, and $\nu_{cm} = 2\pi K/(\xi_H^2\eta)$.
In this case the reference to an effective elastic constant close to the values of the elastic constants in the nematic phase is a reasonable assumption.

Model fits
The total spin-lattice relaxation rate can be expressed as the sum of the three contributions:

$$T_1^{-1} = (T_1^{-1})_{\mathrm{SD}} + (T_1^{-1})_{\mathrm{R}} + (T_1^{-1})_{\mathrm{OF}} \qquad (7)$$

Close to the I-N transition temperature, short-range director fluctuations (DF) were also considered.

The model fit to the experimental results was performed with the open access fitting software *fitteia*® using the least-squares minimization method with a global minimum target *(40)*. Table ST1 lists the fixed parameters used in model fits. Several parameters were obtained independently from previous studies. In the analysis of data for 5CB-αd_2 at different temperatures, Arrhenius temperature dependence of correlation times for rotations and diffusion was assumed, in line with previous research *(28,37)*.

The spin density and the geometric factors estimated for the intra-molecular average inter-proton spin distances used in the fits are presented in Table ST1. The values of viscosity and of self-diffusion coefficients were obtained from the literature except for self-diffusion coefficient of the hexanoic acid, 2-methyl-1-undecene, and n-hexane compounds that were measured by PFG NMR.

Compound	n	$A^{(0)}$	$A^{(1)}$	$A^{(2)}$	η	D
	(10^{28} m^{-6})	(10^{57} m^{-6})	(10^{57} m^{-6})	(10^{58} m^{-6})	(10^{-3} Pa.s)	(10^{-11} m^{2}s^{-1})
5CB-αd_2 (@317K)	4.1	6.3	3.4	0.88	19 *(42)*	8.2 *(43)*
$C_6H_{12}O_2$	5.8	6.5	3.7	1.4	3.1 *(44)*	40
$C_{12}H_{24}$	6.3	6.7	6.5	1.3	~1*	80
C_6H_{14}	6.4	7.3	6.8	1.7	~0.3*	820

Table ST1: Physical parameters fixed in the fits. The uncertainty of these parameters' estimates is less than 2.5%. For 5CB the viscosity in the isotropic phase was obtained from *(42)*, see Figure S5. *Values estimated taking into account the values of η for $C_{11}H_{22}$, $C_{11}H_{24}$, $C_{12}H_{24}$, and $C_{12}H_{26}$ compounds.

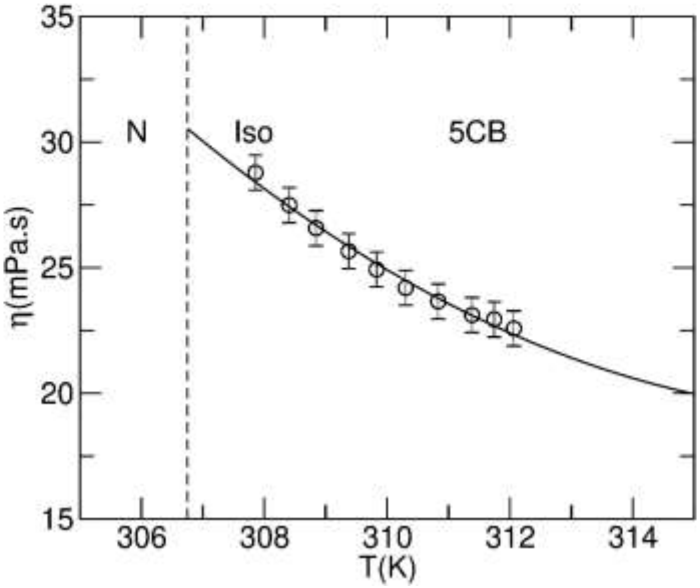

Figure S5: Viscosity data for 5CB obtained from *(42)*. Solid line is a simple phenomenological model equation with $\eta = a + b(T_{ref} - T)^2$; a = 19.1±0.7 mPa s, b = 0.091±0.007 mPa s K^{-2} and T_{ref} = 318 K.

Table ST2 lists the parameters obtained for best fits for relaxation data all four systems shown in Fig 1.

Compound	τ_S (s^{-10} s)	τ_L (s^{-11} s)	$\omega_0/2\pi$ (10^6 Hz)	$\omega_c/2\pi$ (10^6 Hz)	A_{OF} (10^3 $s^{-3/2}$)
5CB-αd_2 (@317K)	14±9	7.8±0.3	4.7±0.5	89±18	20±1
$C_6H_{12}O_2$	3.7±0.3	7.8±0.8	13±2	109±26	4.5±0.3
$C_{12}H_{24}$	1.2±0.1	7.8±0.7	14±3	95±26	1.9±0.2
C_6H_{14}	1.99±0.06	1.99±0.06	-	-	-

Table ST2: Model parameters obtained from the best fits for 5CB-αd_2 at 317K, hexanoic acid, 2-methyl-1-undecene, and n-hexane.

Figure S6 shows the analysis of relaxation data for 5CB-αd_2 at different temperatures. The parameter values for best fits are listed in Table ST3.

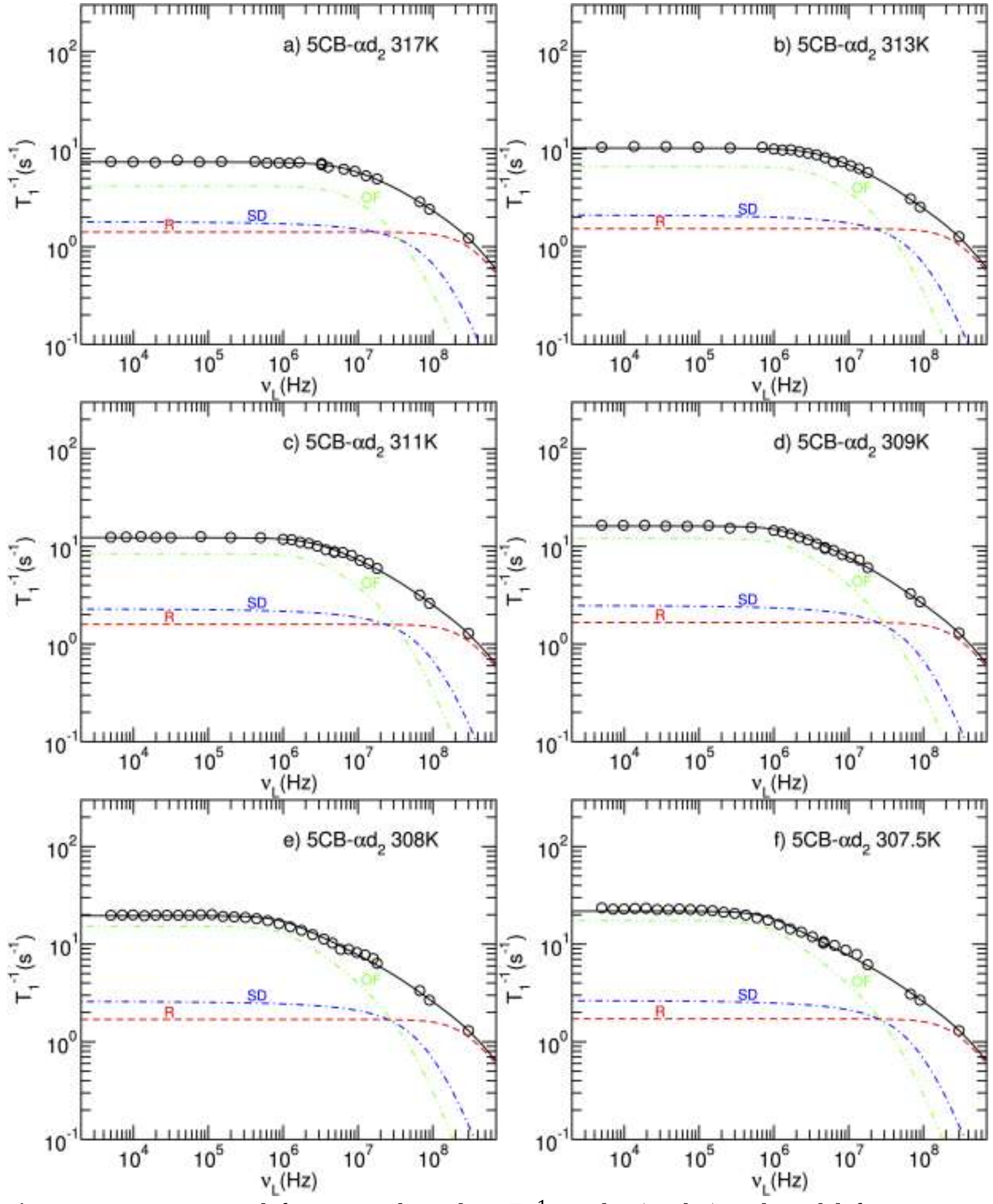

Figure S6: Experimental frequency dependent T_1^{-1} results (circles) and model fitting curves obtained for six temperatures in the isotropic phase for 5CB-αd_2.

T (K)	τ_S (s^{-10} s)	τ_L (s^{-11} s)	$\omega_0/2\pi$ (10^6 Hz)	$\omega_c/2\pi$ (10^6 Hz)	A_{OF} (10^3 $s^{-3/2}$)	D (10^{-11} m^2s^{-1})	η (10^{-3} Pa s)	L (10^{-12} N)	ξ_H (10^{-10} m)
317	14.0	7.8	4.7	89.3	20.0	8.19	19	2.58	21.3
313	14.1	6.5	2.9	79.2	23.2	6.98	21	2.40	24.7
311	13.7	5.9	2.0	76.0	23.4	6.44	24	2.45	28.7
309	13.2	5.4	1.1	74.1	23.2	5.93	26	2.55	37.1
308	13.0	5.1	0.7	73.5	22.2	5.69	28	2.68	47.8
307.5	12.9	5.0	0.4	73.4	20.9	5.57	29	2.81	59.2
307	12.8	4.9	0.2	73.3	16.5	5.46	30	3.33	90.8

Table ST3: Model parameters obtained from the best fits for 5CB-αd_2 at different temperatures. The values of D and η were obtained from literature. L and ξ_H were calculated using Eqs. S5 and S6.

Figure S7 shows the fit obtained for 5CB-αd_2 at 307 K, where we are in the biphasic regime. Therefore, the DF mechanism (Eq. S6) was added to the model. The values of A_{DF}, ν_{cm}, and ν_{cM} were left as free parameters, obtaining $A_{DF} \simeq 1.2\times10^3$ $s^{-3/2}$, $\nu_{cm} \simeq 8$ kHz, while ν_{cM} could not be estimated since its effect on the fit is masked by other relaxation contributions.

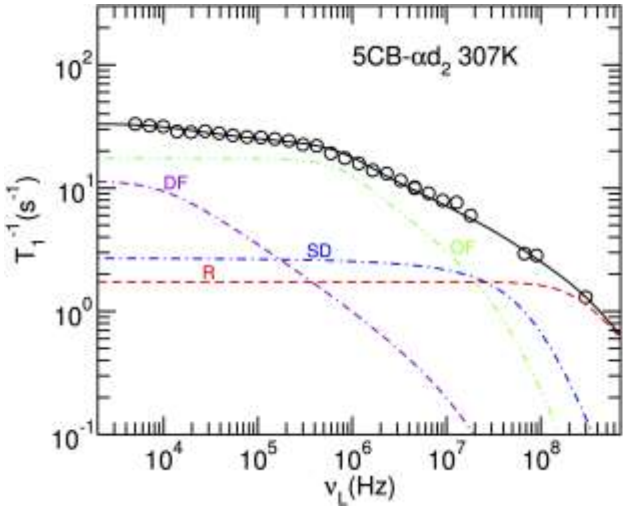

Figure S7: Experimental frequency dependent T_1^{-1} results (circles) and model fitting curves for 5CB-αd_2 at 307 K.

Table ST4 lists the parameters obtained for best fits for X-ray profiles all four systems shown in Fig 1. A sum of Lorentzian curves was used. Bragg's law, $q = 4\pi \sin(\theta)/\lambda$, yields the values of d and ℓ. The coherence lengths $\xi_\perp$, $\xi_\parallel$, and $\widetilde{\xi_\parallel}$ were obtained from the peaks' widths at half- heights, $\Delta_{1/2}$, using the Scherrer formula *(45)* $\Delta_{1/2}(2\theta) \approx K\lambda/(\xi\cos(\theta))$, with $K \approx 0.9$. In terms of wave vectors, for small angles, $\xi \approx 2\pi K/\Delta_{1/2}(q)$.

Compound	d	$\xi_\perp$	ℓ	$\xi_\parallel$	"2ℓ"	$\tilde{\xi}_\parallel$
5CB-αd_2 (nematic), 291 K	4.4±0.1	11±2	15±5	11±5	24±4	53±39
5CB-αd_2 (Isotropic), 317 K	4.5±0.1	10±2	12±5	22±16	27±16	13±4
$C_6H_{12}O_2$, 290 K	4.4±0.1	11±2	-	-	12±1	12±4
$C_{12}H_{24}$, 290 K	4.7±0.1	11±1	-	-	-	-
C_6H_{14}, 290 K	4.6±0.1	10±2		-	-	-

Table ST4: Characteristic distances (in Å) estimated from the X-ray diffraction profiles.

Due to the relatively small intensity of the X-ray diffraction peaks at small q values, the relative uncertainty of the molecular distances and coherence lengths is rather large. For 5CB-αd_2 the anti-parallel arrangement of the molecules can be observed in the X-rays profiles in both nematic and isotropic phases, given that an X-ray peak associated to a distance about "2ℓ" and coherence length $\tilde{\xi}_\parallel$ is detected. This result is consistent with the results obtained by molecular dynamics simulations and reflects the antiparallel arrangement of the electric dipoles associated to the 5CB terminal CN groups *(19,20)*. For hexanoic acid, only a peak with an associated distance $2\ell \sim 12$ Å is detected, which is compatible with the molecular length. The hexanoic acid molecules form dimers and some degree of dimer's stacking is observed from the low-q X-ray peak but the electric charge distribution along the local alignment direction is much more uniform than that observed for 5CB molecular pairs in antiparallel arrangement of the electric dipoles. For the latter the alternance between the bi-phenyl rigid cores and flexible aliphatic chains produces a noticeable additional diffraction peak at lower q values. On the other hand, for the 2-methyl-1-undecene, no stacking is observed, indicated by the absence of the low-q peak. The reason is that even if the molecules locally share the same orientation, there is no local lateral coordination because of the absence of groups that would promote it. In contrast with the 5CB and hexanoic acid molecules, the 2-methyl-1-undecene molecule has a rather uniform electron density distribution.

References

1. Karthika, S., Radhakrishnan, T. K., & Kalaichelvi, P. (2016). A review of classical and nonclassical nucleation theories. *Crystal Growth & Design*, *16*(11), 6663-6681.
2. Vekilov, P. G. (2010). Nucleation. *Crystal growth & design*, *10*(12), 5007-5019.
3. Sleutel, M., Lutsko, J., Van Driessche, A. E., Durán-Olivencia, M. A., & Maes, D. (2014). Observing classical nucleation theory at work by monitoring phase transitions with molecular precision. *Nature communications*, *5*(1), 1-8.

4. Zhang, F. (2017). Nonclassical nucleation pathways in protein crystallization. *Journal of Physics: Condensed Matter*, *29*(44), 443002.
5. Wolde, P. R. T., & Frenkel, D. (1997). Enhancement of protein crystal nucleation by critical density fluctuations. *Science*, *277*(5334), 1975-1978.
6. Ou, Z., Wang, Z., Luo, B., Luijten, E., & Chen, Q. (2020). Kinetic pathways of crystallization at the nanoscale. *Nature materials*, *19*(4), 450-455.
7. Erdemir, D., Lee, A. Y., & Myerson, A. S. (2009). Nucleation of crystals from solution: classical and two-step models. *Accounts of chemical research*, *42*(5), 621-629.
8. Yang, J., Koo, J., Kim, S., Jeon, S., Choi, B. K., Kwon, S., ... & Park, J. (2019). Amorphous-phase-mediated crystallization of Ni nanocrystals revealed by high-resolution liquid-phase electron microscopy. *Journal of the American Chemical Society*, *141*(2), 763-768.
9. de Gennes, P. G., Prost, J. *The Physics of Liquid Crystals*. (Oxford University Press, 1993).
10. Blinc, R., Hogenboom, D. L., O'Reilly, D. E. & Peterson, E. M. Spin Relaxation and Self-Diffusion in Liquid Crystals. *Phys. Rev. Lett.* **23**, 969 (1969).
11. Ghosh, S. K., Tettamanti, E. & Indovina, P. L. Dynamical behavior of a nematic liquid crystal just above the nematic-isotropic transition from spin-lattice relaxation. *Phys. Rev. Lett.* **29**, 638 (1972).
12. Dong, R. Y., Tomchuk, E. & Bock, E. Proton Spin Relaxation Study of Order Fluctuations Above the Nematic--Isotropic Transition in the Liquid Crystal MBBA: II. Coherent and Incoherent Scattering. *Can. J. Phys.* **53**, 610–616 (1975).
13. Cabane, B. & Clark, W. G. Effects of Order and Fluctuations on the N 14 NMR in a Liquid Crystal. *Phys. Rev. Lett.* **25**, 91 (1970).

14. Visintainer, J. J., Bock, E., Dong, R. Y. & Tomchuk, E. A proton FFT study of the aromatic and alkyl motions in the liquid crystal CBOOA: Orientational order fluctuations in the isotropic phase. *Can. J. Phys.* **54**, 2282–2286 (1976).
15. Val'kov, A. Y., Romanov, V. P. & Shalaginov, A. N. Fluctuations and light scattering in liquid crystals. *Physics-Uspekhi* **37**, 139–183 (1994).
16. Dong, R. Y., Tomchuk, E., Visintainer, J. J. & Bock, E. 14N NMR Study of Order Fluctuations in the Isotropic Phase of Liquid Crystals. *Mol. Cryst. Liq. Cryst.* **33**, 101–111 (1976).
17. Stinson, T. W. & Litster, J. D. Pretransitional Phenomena in the Isotropic Phase of a Nematic Liquid Crystal. Phys. Rev. Lett. 25, 503 (1970).
18. Stinson, T. W. & Litster, J. D. Correlation Range of Fluctuations of Short-Range Order in the Isotropic Phase of a Liquid Crystal. Phys. Rev. Lett. 30, 688 (1973).
19. Zhang, J., Su, J. & Guo, H. An Atomistic Simulation for 4-Cyano-4'-pentylbiphenyl and Its Homologue with a Reoptimized Force Field. *J. Phys. Chem. B* **115**, 2214–2227 (2011).
20. Palermo, M. F., Pizzirusso, A., Muccioli, L. & Zannoni, C. An atomistic description of the nematic and smectic phases of 4-n-octyl-4' cyanobiphenyl (8CB). *J. Chem. Phys.* **138**, 204901 (2013).
21. Cook, M. J. & Wilson, M. R. Simulation studies of dipole correlation in the isotropic liquid phase. *Liq. Cryst.* **27**, 1573–1583 (2000).
22. Angell, C. A. & Zhao, Z. Fluctuations, clusters, and phase transitions in liquids, solutions, and glasses: from metastable water to phase change memory materials. *Faraday Discuss.* **167**, 625–641 (2014).

23. Malm, A. V., & Corbett, J. C. (2019). Improved dynamic light scattering using an adaptive and statistically driven time resolved treatment of correlation data. *Scientific reports*, *9*(1), 1-11.

24. De Jonge, N., & Ross, F. M. (2011). Electron microscopy of specimens in liquid. *Nature nanotechnology*, *6*(11), 695-704.

25. Dong, R. Y. *Nuclear magnetic resonance of liquid crystals*. (Springer New York, 1997).

26. Abragam, A. *The principles of nuclear magnetism*. (Oxford university press, 1961).

27. Kimmich, R. Field-cycling NMR Relaxometry: Instrumentation, Model Theories and Applications. *R. Soc. Chem.* 358–384 (2018).

28. Sebastião, P. J., Gradišek, A., Pinto, L. F. V., Apih, T., Godinho, M. H., & Vilfan, M. Fast field-cycling NMR relaxometry study of chiral and nonchiral nematic liquid crystals. *J. Phys. Chem. B* **115**, 14348-14358, (2011).

29. Gradišek, A., Sebastião, P. J., Fernandes, S. N., Apih, T., Godinho, M. H., & Seliger, J. 1H-2H cross-relaxation study in a partially deuterated nematic liquid crystal. *J. Phys. Chem. B* **118**, 5600–5607 (2014).

30. Bunning, J. D., Faber, T. E. & Sherrell, P. L. The Frank constants of nematic 5CB at atmospheric pressure. *J. Phys.* **42**, 1175–1182 (1981).

31. Nuclear Magnetic Resonance Spectroscopy of Liquid Crystals, pp. 129-167, Ed. R. Dong, World Scientific Co., 2009

32. Zhang, J., Ferraz, A., Ribeiro, A. C., Sebastiăo, P. J., & Dong, R. Y. (2006). Deuterium nuclear-magnetic-resonance study of a chiral smectic-C* phase. *Physical Review E, 74*(6), 061704.

33. Anderson, P. W. (1972). More is different: broken symmetry and the nature of the hierarchical structure of science. *Science*, *177*(4047), 393-396.

34. Anderson, P. W., & Stein, D. L. (1987). Broken symmetry, emergent properties, dissipative structures, life. In Self-organizing systems (pp. 445-457). Springer, Boston, MA.

35. Mesquita Sousa, D., Domingos Marques, G., Manuel Cascais, J. & José Sebastião, P. (2010). Desktop fast-field cycling nuclear magnetic resonance relaxometer. *Solid State Nucl. Magn. Reson.* **38**, 36–43.

36. Gane PAC, Leadbetter AJ, Benattar JJ, et al. Structural correlations in smectic-F and smectic-I phases. *Phys Rev A* **24**, 2694–2700, (1981).

37. Apih, T., Domenici, V., Gradišek, A., Hamplova, V., Kaspar, M., Sebastião, P. J., & Vilfan, M. ^{1}H NMR relaxometry study of a rod-like chiral liquid crystal in its isotropic, cholesteric, TGBA*, and TGBC* phases. *J. Phys. Chem. B* **114**, 11993-12001, (2010).

38. Gradišek, A., Apih, T., Domenici, V., Novotna, V. & Sebastião, P. J. Molecular dynamics in a blue phase liquid crystal: A 1H fast field-cycling NMR relaxometry study. *Soft Matter* **9**, 10746-10753, (2013).

39. Gradišek, A., Domenici, V., Apih, T., Novotná, V. & Sebastião, P. J. ^{1}H NMR Relaxometric Study of Molecular Dynamics in a 'de Vries' Liquid Crystal. *J. Phys. Chem. B* **120**, 4706-4714, (2016).

40. Torrey, H. C. Nuclear Spin Relaxation by Translational Diffusion. *Phys. Rev.* **92**, 962 (1953).

41. Sebastião, P. J. The art of model fitting to experimental results. *Eur. J. Phys.* **35**, 015017 (2013).

42. Chmielewski, A. G. Viscosity Coefficients of Some Nematic Liquid Crystals. *Mol. Cryst.*

Liq. Cryst. **132**, 339–352 (1986).

43. Dvinskikh, S. V, Furo, I., Zimmermann, H. & Maliniak, A. Anisotropic self-diffusion in thermotropic liquid crystals studied by H-1 and H-2 pulse-field-gradient spin-echo NMR. *Phys. Rev. E* **65**, 61701 (2002).
44. Ghatee, M. H. *et al.* Molecular dynamics simulation and experimental approach to the temperature dependent surface and bulk properties of hexanoic acid. *Ind. Eng. Chem. Res.* **52**, 3334–3341 (2013).
45. Patterson, A. L. The Scherrer Formula for X-Ray Particle Size Determination. *Phys. Rev.* **56**, 978 (1939).
